# Potential Effects of Metformin on the Vitality, Invasion, and Migration of Human Vascular Smooth Muscle Cells via Downregulating lncRNA-ATB

**DOI:** 10.1155/2022/7480199

**Published:** 2022-01-04

**Authors:** Wei Jia, Yue Zhou, Lina Sun, Jianlong Liu, Zhiyuan Cheng, Shuofang Zhao

**Affiliations:** ^1^Department of Vascular Surgery, Beijing Jishuitan Hospital, Beijing, China; ^2^Department of Neurological Rehabilitation, Weifang Yidu Central Hospital, Weifang, China; ^3^Emergency Center, Qingdao Central Hospital Affiliated to Qingdao University, Qingdao, China; ^4^Guangdong Academy of Medical Sciences and Guangdong Provincial People's Hospital, Guangzhou, China

## Abstract

**Objective:**

To elucidate the role of metformin in influencing VSMCs via the involvement of lncRNA-ATB.

**Methods:**

qRT-PCR was conducted to detect serum levels of lncRNA-ATB and p53 in CHD patients (*n* = 50) and healthy subjects (*n* = 50). Correlation in serum levels of lncRNA-ATB and p53 in CHD patients was assessed by Pearson correlation test. ROC curves were depicted for analyzing the predictive potential of lncRNA-ATB in the occurrence of CHD. After metformin induction in VSMCs overexpressing lncRNA-ATB, relative levels of lncRNA-ATB and p53 were detected. Meanwhile, proliferative, migratory, and invasive abilities in VSMCs were, respectively, examined by CCK-8 and transwell assay. The interaction between lncRNA-ATB and p53 was tested by RIP. In addition, the coregulation of lncRNA-ATB and p53 in cell functions of VSMCs was finally determined.

**Results:**

Increased serum level of lncRNA-ATB and decreased p53 level were detected in CHD patients than those of healthy subjects. LncRNA-ATB could interact with p53 and negatively regulate its level. In addition, lncRNA-ATB could serve as a potential biomarker for predicting the occurrence of CHD. The overexpression of lncRNA-ATB triggered viability, migratory, and invasive abilities in VSMCs, and the above trends were abolished by metformin induction. The overexpression of p53 partially abolished the promotive effects of lncRNA-ATB on proliferative, migratory, and invasive abilities in VSMCs.

**Conclusions:**

Metformin induction inhibits proliferative, migratory, and invasive abilities in VSMCs by downregulating lncRNA-ATB, which may be related to p53 activation.

## 1. Introduction

Vascular smooth muscle cells (VSMCs) are the main cell types of vascular wall components. They are mainly located in the vascular mesothelium for maintaining blood vessel tension and regulating blood pressure [1]. Abnormal proliferation and migration of VSMCs are the fundamental pathological basis for atherosclerosis, hypertension, and restenosis after coronary artery intervention and other vascular proliferative diseases [2].

Metformin is a widely applied drug for type 2 diabetes mellitus. In addition, it is useful in preventing against atherosclerosis. A clinical trail involving 3,234 prodromal diabetes subjects showed that compared with the placebo group, the incidence and severity of coronary artery calcification remarkably decrease in those treated by metformin [3]. It is suggested that metformin can prevent diabetes-induced atherosclerosis. Sainio et al. [4] also suggested the long-term protection of metformin on blood vessels.

LncRNAs are transcripts containing more than 200 nucleotides and they cannot be transcribed into proteins [5]. They are involved in the regulation of atherosclerosis progression [6]. LncRNA-ATB is the first lncRNA that can be activated by TGF-*β*, which is located on chromosome 14 [7]. It is vital regulator involved in phenotype transformation of human peritoneal mesothelial cells, hepatitis C-related cirrhosis, and preeclampsia [8–10]. Yue et al. [11] demonstrated that the upregulated lncRNA-ATB is an oncogene involved in the progression of colorectal cancer. Shi et al. [12] uncovered that lncRNA-ATB drives trastuzumab resistance and invasion-metastasis cascade. Currently, the potential influences of lncRNA-ATB on cell functions of VSMCs and progression of vascular diseases remain unclear.

A previous study has elucidated the biological function of metformin in regulating bladder cancer cell proliferation and glycolysis [13]. In addition, metformin is reported to inhibit proliferative and migratory potentials in human primary VSMCs [14]. However, whether lncRNA-ATB is involved in metformin-induced VSMCs behaviors is unknown, which is specifically explored in this paper.

## 2. Patients and Methods

### 2.1. Case Collection

CHD (coronary heart disease) patients (*n* = 50) pathologically confirmed by coronary angiography in Beijing Jishuitan Hospital were recruited. During the same period, 50 healthy subjects undergoing physical examinations were recruited in the control group. Inclusion criteria of CHD patients were as follows: stable angina patients with at least one main coronary artery stenosis > 80%. Exclusion criteria is as follows: (i) unstable angina or myocardial infarction patients; (ii) combined with other organic heart diseases; and (iii) combined with severe liver and kidney diseases, familial hypercholesterolemia, malignant tumors, or inflammatory diseases. 3 mL of venous blood was extracted from each subject. Sample collection was reviewed and approved by the hospital ethics committee, and every subject signed written informed consent.

### 2.2. Cell Culture of VSMCs

Human VSMCs were purchased from the National Infrastructure of Cell Line Resource. Cells were quickly taken out from liquid nitrogen tank and subjected to water bath at 37°C. Cells were suspended in 5-10 times the volume of Roswell Park Memorial Institute 1640 (RPMI 1640) (HyClone, South Logan, UT, USA) and centrifuged at 160 × g for 5 min. The precipitant was resuspended in 1 mL of RPMI-1640 containing 10% fetal bovine serum (FBS) (Gibco, Rockville, MD, USA) for cell culture in a 5% CO_2_ incubator at 37°C. VSMCs were induced with 10 mmol/L metformin for 24 h.

### 2.3. Cell Transfection

Overexpression plasmids and siRNAs were, respectively, provided by Genomeditech (Shanghai, China) and Ribobio (Guangzhou, China). 2 × 10^5^ cells per well were inoculated in 6-well plates and cultured to 80% confluence. Subsequently, 1,750 *μ*L of serum-free medium was replaced per well. Transfection plasmids (2 *μ*g) and Lipofectamine 3000 (5 *μ*L) (Invitrogen, Carlsbad, CA, USA) were, respectively, diluted in 125 *μ*L of serum-free medium and let stand at room temperature for 5 min, followed by mixture of two solutions. The mixture was applied in each well. Fresh medium containing 10% FBS was replaced at 5 h.

### 2.4. Quantitative Real-Time Polymerase Chain Reaction (qRT-PCR)

RNA extraction kit (ABI, Foster City, CA, USA) was used for extracting total RNAs from tissues or cells, and their concentration and purity were measured by an ultraviolet spectrophotometer (Thermo Fisher, Waltham, MA, USA). Reverse transcription in a 20 *μ*L system was conducted to obtain complementary deoxyribose nucleic acid (cDNA), which was subjected to qRT-PCR at 95°C predenaturation for 1 min, 40 cycles at 95°C for 15 s, and 60°C for 1 min. Relative levels of lncRNA-ATB, p53, and glyceraldheyde 3-phosphate dehydrogenase (GAPDH) were calculated by 2^-*ΔΔ*Ct^. GAPDH was the internal reference. Primer sequences were as follows: lncRNA-ATB forward, 5′-CTTCACCAGCACCCAGAGA-3′ and reverse, 5′-AAGACAGAAAAACAGTTCCGAGTC-3′; p53 forward, 5′-TCAGTCTACCTCCCGCCATA-3′ and reverse, 5′-TTACATCTCCCAAACATCCCT-3′; GADPH forward, 5′-GGTGAAGGTCGGAGTCAACG-3′ and reverse, 5′-CAAAGTTGTCATGGATGHACC-3′.

### 2.5. Cell Counting Kit-8 (CCK-8)

Cells were inoculated in the 96-well plates at the density of 3 × 10^3^ cells/well. Six replicates were prepared in each group. At day 0, 1, 2, and 3, 10 *μ*L of CCK-8 solution (Dojindo, Kumamoto, Japan) was used for 1 h incubation, and optical density at 450 nm was measured.

### 2.6. Transwell Assay

Transwell chambers (8 *μ*m) containing 200 *μ*L of serum-free suspension (2 × 10^4^ cells) were inserted in the 24-well plate where 600 *μ*L of medium containing 10% FBS was applied in each well. After 24 h cell culture, transwell chambers were taken out. Cells in the bottom were subjected to methanol fixation for 15 min and crystal violet staining for 20 min. Migratory cells were counted in 5 randomly selected fields per sample. Transwell invasion assay was conducted using chambers that were precoated with 50 *μ*L of diluted Matrigel (dilution in serum-free medium at 1 : 7).

### 2.7. RNA Binding Protein Immunoprecipitation Assay (RIP)

EZMagna RIP kit (Millipore, Billerica, MA, USA) was used. Cells were lysed in RIPA and incubated at 4°C for 6 hours with magnetic beads conjugated with anti-Ago2 or anti-IgG. Subsequently, magnetic beads were washed and incubated with Protease K for removing proteins. The purified RNA was subjected to qRT-PCR.

### 2.8. Statistical Analysis

Statistical Product and Service Solutions (SPSS) 20.0 (IBM, Armonk, NY, USA) was adopted for statistical processing. Correlation in serum levels of lncRNA-ATB and p53 in CHD patients was assessed by Pearson correlation test. ROC curves were depicted for analyzing the potential of lncRNA-ATB in predicting the occurrence of CHD. Differences between groups were analyzed by the Student's *t*-test. A significant difference was set at *p* < 0.05.

## 3. Results

### 3.1. Serum Level of lncRNA-ATB Increases in CHD Patients

**Figure 1:**
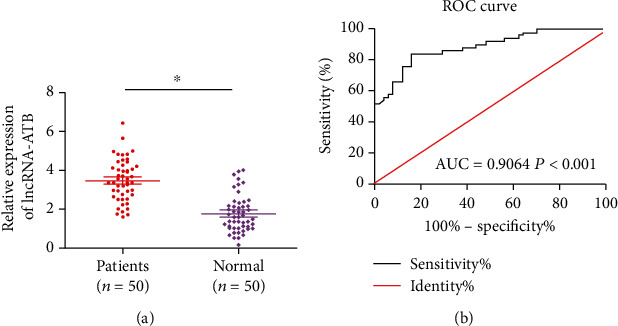
Serum level of lncRNA-ATB increases in CHD patients. (a) Serum level of lncRNA-ATB was higher in CHD patients than healthy subjects. (b) LncRNA-ATB could be used as a potential biomarker for predicting the occurrence of CHD.

Compared with healthy subjects, serum level of lncRNA-ATB was higher in CHD patients (Figure 1(a)). To ascertain the potential of lncRNA-ATB as a biomarker for predicting the occurrence of CHD, ROC curves were depicted. The AUC was 0.9064, indicating that lncRNA-ATB exerted a certain diagnostic potential in CHD (*p* < 0.001, Figure 1(b)).

### 3.2. LncRNA-ATB Triggers Viability, Migratory, and Invasive Abilities in VSMCs

**Figure 2:**
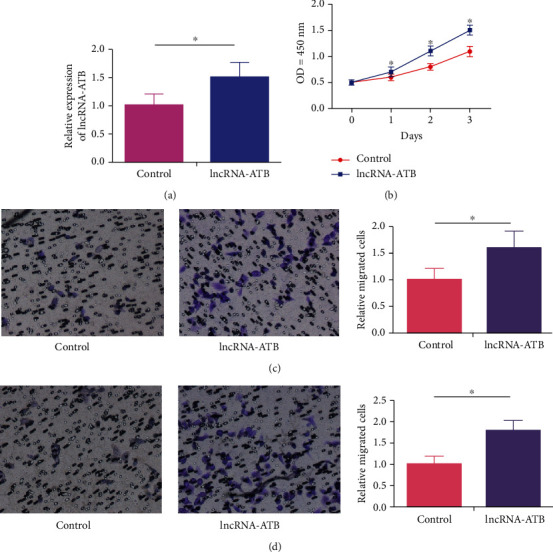
LncRNA-ATB triggers viability, migratory, and invasive abilities in VSMCs. (a) Transfection of pcDNA-lncRNA-ATB effectively upregulated lncRNA-ATB in VSMCs. (b) CCK-8 assay showed dose-dependently increased viability in VSMC overexpressing lncRNA-ATB. (c) Transwell assay showed enhanced migration in VSMC overexpressing lncRNA-ATB. (d) Transwell assay showed enhanced invasion in VSMC overexpressing lncRNA-ATB.

In vitro experiments were conducted in VSMCs to elucidate the potential role of lncRNA-ATB in the progression of CHD. Transfection of pcDNA-lncRNA-ATB remarkably upregulated lncRNA-ATB in VSMCs, verifying the pronounced transfection outcome (Figure 2(a)). The overexpression of lncRNA-ATB time-dependently enhanced cell viability in VSMCs (Figure 2(b)). Moreover, transwell assay showed that the overexpression of lncRNA-ATB increased the rates of migratory and invasive cells in VSMCs, indicating the stimulated metastasis ability (Figures 2(c) and 2(d)).

### 3.3. Metformin Inhibits Viability, Migratory, and Invasive Abilities in VSMCs

**Figure 3:**
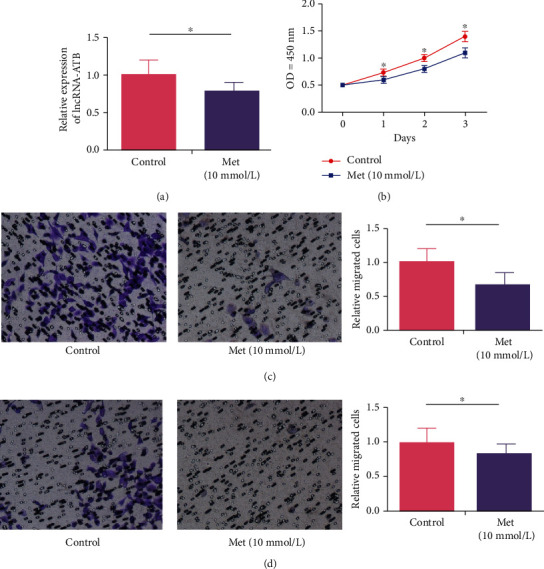
Metformin inhibits viability, migratory, and invasive abilities in VSMCs. (a) LncRNA-ATB was downregulated by metformin induction in VSMCs. (b) CCK-8 assay showed dose-dependently increased viability in metformin-induced VSMCs. (c) Transwell assay showed enhanced migration in metformin-induced VSMCs. (d) Transwell assay showed enhanced invasion in metformin-induced VSMCs.

We subsequently explored the potential influence of metformin on cell functions of VSMCs. After 10 mmol/L metformin induction for 24 h, lncRNA-ATB was markedly downregulated in VSMCs (Figure 3(a)). Besides, metformin induction reduced viability and the rates of migratory and invasive cells in VSMCs (Figures 3(b) and 3(d)).

### 3.4. LncRNA-ATB Inhibits p53 Level

**Figure 4:**
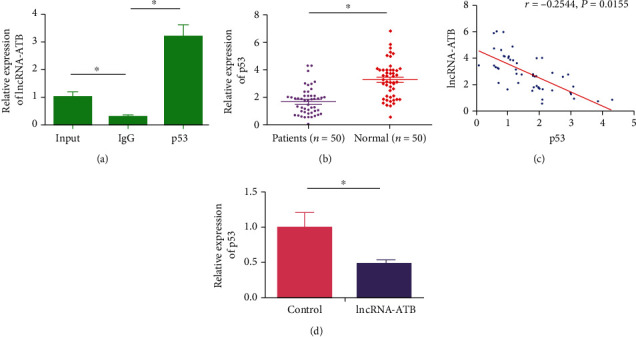
LncRNA-ATB inhibits p53 level. (a) RIP assay showed that lncRNA-ATB interacted with p53. (b) Serum level of p53 was lower in CHD patients than healthy subjects. (c) Pearson correlation analysis showed a negative correlation between serum levels of lncRNA-ATB and p53 in CHD patients. (d) Overexpression of lncRNA-ATB downregulated p53 in VSMCs.

RIP assay showed that lncRNA-ATB was mainly enriched in anti-p53, indicating the interaction between lncRNA-ATB and p53 (Figure 4(a)). Compared with healthy subjects, serum level of p53 was lower in CHD patients (Figure 4(b)). Moreover, serum level of lncRNA-ATB was negatively correlated to that of p53 in CHD patients (*r* = −0.2544, *p* = 0.0155, Figure 4(c)). As expected, the overexpression of lncRNA-ATB could downregulate p53 in VSMCs, further supporting their negative interaction (Figure 4(d)). The above data demonstrated that lncRNA-ATB was negatively interacted with p53, and they were both involved in CHD progression.

### 3.5. Regulatory Effects of lncRNA-ATB on VSMCs Require the Interaction with p53

**Figure 5:**
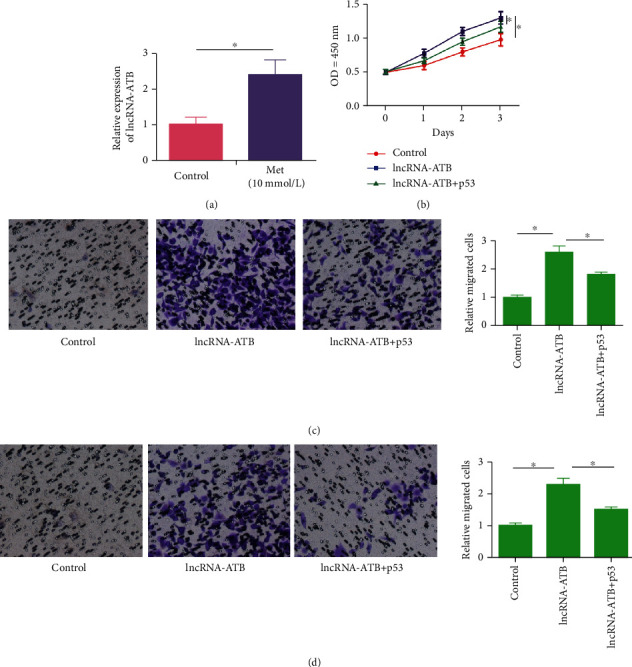
Regulatory effects of lncRNA-ATB on VSMCs require the interaction with p53. (a) P53 was upregulated by metformin induction in VSMCs. (b) Overexpression of p53 abolished the role of lncRNA-ATB in promoting VSMC proliferation. (c) Overexpression of p53 abolished the role of lncRNA-ATB in promoting VSMCs migration. (d) Overexpression of p53 abolished the role of lncRNA-ATB in promoting VSMC invasion.

To further verify that both lncRNA-ATB and p53 were involved in the regulatory mechanism of metformin on VSMCs, p53 level in metformin-induced VSMCs was detected by qRT-PCR, which was remarkably upregulated (Figure 5(a)). It is suggested that metformin could stimulate p53 activation. CCK-8 assay uncovered that the overexpression of p53 in VSMCs could reverse the effect of lncRNA-ATB on stimulating VSMC proliferation (Figure 5(b)). Identically, p53 also abolished the promotive effects of highly expressed lncRNA-ATB on VSMC metastasis (Figures 5(c) and 5(d)). To sum up, metformin activated the p53 expression by downregulating lncRNA-ATB, thereafter inhibiting proliferative, migratory, and invasive abilities in VSMCs.

## 4. Discussion

VSMCs are responsible for regulating blood flow, balancing vessel wall elasticity, and tension [15, 16]. Under the normal circumstance, VSMCs lack the abilities to proliferate and migrate. Once vascular endothelium damages, VSMCs rapidly acquire the capacities of proliferation, metastasis, and release of extracellular matrix under the stimuli of multiple molecules [17]. Marx et al. [18] and Uemura et al. [19] uncovered that the main component of angiogenesis intima is the migrated VSMCs from the tunica media, and they will continue to proliferate in the neointima. Overproliferated VSMCs will develop to vessel wall thickening and lumen stenosis. Excessive proliferated and migrated VSMCs are almost involved in every process of intimal neoplasia, atherosclerosis, and restenosis [20, 21]. Therefore, inhibition of overproliferated VSMCs contributes to alleviate the progression of atherosclerosis.

A growing number of studies have reported that lncRNAs are able to regulate cell functions of VSMCs. LncRNA TUG1 induces hypertension progression via mediating proliferative and migratory abilities in VSMCs [22]. LncRNA-ATB is activated by TGF-*β*, with the transcription length of 2.4 kb [23]. In renal cell carcinoma (RCC) samples, lncRNA-ATB is upregulated, especially metastatic ones. Silence of lncRNA-ATB by siRNA transfection remarkably blocks EMT and induces apoptosis [24]. Furthermore, high level of lncRNA-ATB predicts poor prognosis in RCC patients as an independent prognostic factor [25]. Consistently, our analysis showed higher serum level of lncRNA-ATB in CHD patients, and lncRNA-ATB may become a potential diagnostic factor for CHD. In vitro experiments subsequently proved that lncRNA-ATB was able to stimulate VSMCs to proliferate and metastasize. Meanwhile, metformin induction time-dependently decreased viability in VSMCs overexpressing lncRNA-ATB. We believed that metformin inhibited VSMC viability by downregulating lncRNA-ATB.

P53 is a widely analyzed transcription factor, which is capable of regulating cell cycle progression, cell apoptosis, autophagy, and other cell functions [26, 27]. Tripathi et al. [28] found out that p53 activity is weakened by silencing MALAT1 in HeLa, U2OS, and WI-38-VA13 cancer cell lines, suggesting that p53 is the downstream target of MALAT1. Our findings revealed a decline in p53 level by the overexpression of lncRNA-ATB in VSMCs. In previous studies, lncRNA usually bind to the RNA binding protein thus to regulate the expression level of target genes. In our research, we find that ATB could directly bind to p53 and thus regulate the expression level. In a recent study, LINC01554 maintained the high G3BP2 expression in ESCC by protecting G3BP2 from degradation through ubiquitination also edified us the mechanism that lncRNA could bind to target gene and regulate that [29]. In addition, metformin induction remarkably upregulated p53 in VSMCs. Based the above results, it is speculated that metformin activated p53 by downregulating lncRNA-ATB, thereby exerting its inhibitory effects on proliferative, migratory and invasive abilities in VSMCs. In summary, this research slightly uncovers the interaction of metformin, ATB, and p53 by in vitro assay. However, the in vivo assay should be done to deeply explore the role of ATB in CHD.

## 5. Conclusion

Metformin induction inhibits proliferative, migratory, and invasive abilities in VSMCs by downregulating lncRNA-ATB, which may be related to p53 activation. This study provides innovative insights into the treatment of vascular diseases.

## Data Availability

The datasets used and analyzed during the current study are available from the corresponding author on reasonable request.
